# Evolutionary analysis of the cystatin family in three *Schistosoma* species

**DOI:** 10.3389/fgene.2014.00206

**Published:** 2014-07-09

**Authors:** Yesid Cuesta-Astroz, Larissa L. S. Scholte, Fabiano Sviatopolk-Mirsky Pais, Guilherme Oliveira, Laila A. Nahum

**Affiliations:** ^1^Grupo de Genômica e Biologia Computacional, Centro de Excelência em Bioinformática, Instituto Nacional de Ciência e Tecnologia em Doenças Tropicais, Centro de Pesquisas René Rachou (CPqRR), Fundação Oswaldo Cruz (Fiocruz)Belo Horizonte, Brazil; ^2^Departamento de Bioquímica e Imunologia, Instituto de Ciências Biológicas, Universidade Federal de Minas GeraisBelo Horizonte, Brazil; ^3^Faculdade Infórium de TecnologiaBelo Horizonte, Brazil

**Keywords:** schistosomiasis, proteinase inhibitor, phylogenomics, bayesian inference, function prediction

## Abstract

The cystatin family comprises cysteine protease inhibitors distributed in 3 subfamilies (I25A–C). Family members lacking cystatin activity are currently unclassified. Little is known about the evolution of *Schistosoma* cystatins, their physiological roles, and expression patterns in the parasite life cycle. The present study aimed to identify cystatin homologs in the predicted proteome of three *Schistosoma* species and other Platyhelminthes. We analyzed the amino acid sequence diversity focused in the identification of protein signatures and to establish evolutionary relationships among *Schistosoma* and experimentally validated human cystatins. Gene expression patterns were obtained from different developmental stages in *Schistosoma mansoni* using microarray data. In *Schistosoma*, only I25A and I25B proteins were identified, reflecting little functional diversification. I25C and unclassified subfamily members were not identified in platyhelminth species here analyzed. The resulting phylogeny placed cystatins in different clades, reflecting their molecular diversity. Our findings suggest that *Schistosoma* cystatins are very divergent from their human homologs, especially regarding the I25B subfamily. *Schistosoma* cystatins also differ significantly from other platyhelminth homologs. Finally, transcriptome data publicly available indicated that I25A and I25B genes are constitutively expressed thus could be essential for schistosome life cycle progression. In summary, this study provides insights into the evolution, classification, and functional diversification of cystatins in *Schistosoma* and other Platyhelminthes, improving our understanding of parasite biology and opening new frontiers in the identification of novel therapeutic targets against helminthiases.

## Introduction

Five species of the genus *Schistosoma* (Trematoda) are involved in the human infection, being the main etiologic agents of human schistosomiasis: *Schistosoma mansoni* and *Schistosoma japonicum* causing intestinal schistosomiasis, and *Schistosoma haematobium* causing urinary schistosomiasis. According to the World Health Organization, schistosomiasis is endemic in 77 countries, affects more than 200 million people worldwide, and other 779 million live in areas at risk of infection (WHO, 2012). Schistosomiasis control relies mainly on praziquantel treatment but its efficacy is limited. Furthermore, evidence of praziquantel resistant parasites was obtained in the laboratory and in endemic regions (Liang et al., 2003; Melman et al., 2009; Coeli et al., 2013). Hence schistosomiasis is still one of the most prevalent infectious and parasitic diseases worldwide being a major source of morbidity and mortality in developing countries.

The urgent need to develop novel drugs or a vaccine for *Schistosoma* species has encouraged an interest in the function prediction of relevant proteins for parasitism. The search for new drug targets based on evolutionary analyses using *S. mansoni* genomic/proteomic data has been performed (Silva et al., 2011, 2012). Such studies have improved the *S. mansoni* functional annotation, allowed for a deeper understanding of the genomic complexity and lineage-specific adaptations potentially related to the parasitic lifestyle, and pointed out several proteins as potential drug targets, including proteases.

Cysteine proteases, one of the four major classes of proteolytic enzymes, have been found in a wide range of taxonomic groups, from viruses to vertebrates. These peptidases are involved in many biological processes, such as catabolism, antigen processing, inflammation, dystrophy, and metastasis (Henskens et al., 1996). Protease inhibitors, such as cystatins, inhibit the enzymatic activity of cysteine proteases. Cystatins comprise a family of cysteine protease inhibitors identified in diverse taxonomic groups, including Platyhelminthes and Nematoda (Kordis and Turk, 2009). In humans, cystatins have evolved widely not only to regulate enzymes in pathways but also as a defense mechanism against proteases of invading pathogens (Toh et al., 2010). In parasites, cystatins participate in normal physiological processes, but are also important pathogenicity factors, being directly involved in host-parasite interactions (Hartmann et al., 1997; Manoury et al., 2001; Schierack et al., 2003; Harnett, 2014).

**Figure 1:**
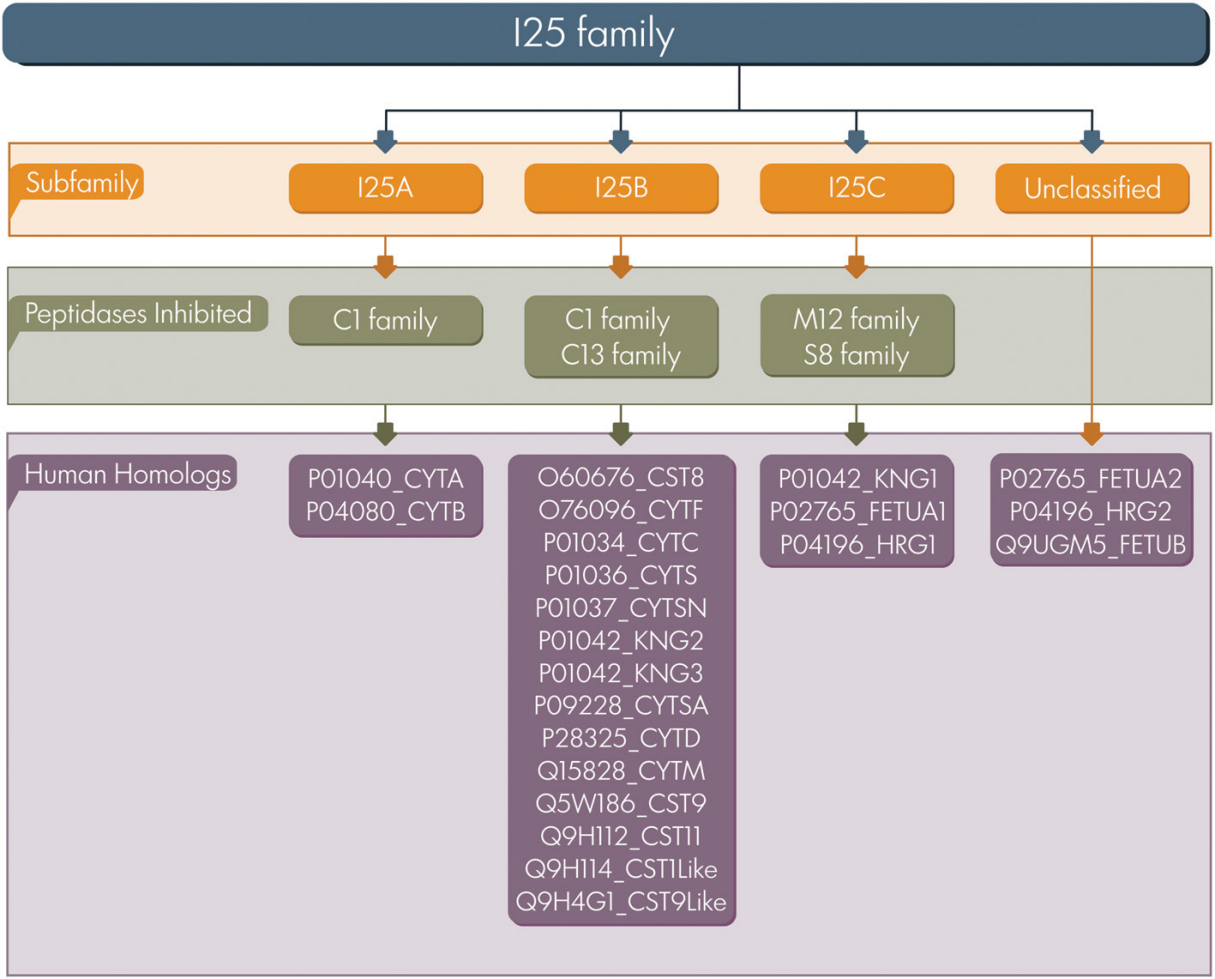
**Cystatin classification**. The I25 family is classified in three subfamilies (I25A–C) according to MEROPS. Several family members remain unclassified. UniProt accession numbers of human homologs are listed.

Based on sequence similarity, the presence or lack of disulfide bonds, and physiological localization, cystatins were first classified in three families: family 1 (e.g., stefins), family 2 (e.g., cystatins), and family 3 (e.g., kininogens) (Barrett, 1986). Afterwards, in terms of number of cystatin domains and the presence of sequence features these proteins were classified into type 1, 2, and 3 (Rawlings and Barrett, 1990). In the present study we adopted the classification proposed by MEROPS database a resource for peptidases and protein inhibitors (Rawlings et al., 2014). The database uses a hierarchical structure-based classification in which each peptidase and inhibitor amino acid sequences are grouped into families based on statistically significant similarities. MEROPS classifies cystatin proteins as members of the I25 family, further subdivided into four subfamilies: I25A, I25B, I25C, and unclassified (Figure 1). This classification system is based on similarities between protein sequences and three dimensional structures. According to MEROPS classification, proteins containing a single inhibitor unit are termed simple inhibitor, and those containing multiple inhibitor units are termed as a compound inhibitor (Rawlings et al., 2014). However, several proteins containing cystatin domains cannot be easily included in a classification scheme, resulting in a number of cystatin family members that remain without classification in the subfamily level (Cornwall et al., 2003; Kordis and Turk, 2009; Siricoon et al., 2012).

One of the first cystatin proteins described in parasitic organisms was the onchocystatin (I25B), a highly antigenic protein encoded by the nematode *Onchocerca volvulus* (Lustigman et al., 1991, 1992). Onchocystatin was initially proposed to be involved in parasite protease regulation during the molting process in Nematoda. Afterwards, it was shown that this protein is also involved in modulation of host immune responses (Hartmann et al., 1997). The molecular interactions of parasite cystatins and host molecules have not yet been clearly determined, but it is believed that the mechanisms are similar to those demonstrated for other species (Klotz et al., 2011). Some examples of known host parasite interactions were previously described in nematodes in which I25B secreted cystatins inhibit host cathepsins such as B and H by *Haemonchus contortus* (Newlands et al., 2001), L and S by *Acanthocheilonema viteae* (Vray et al., 2002), and B and L by *Nippostrongylus brasiliensis* (Dainichi et al., 2001).

Although cystatin family members have been the subject of many studies in different organisms, little is known regarding functional diversification and evolution in *Schistosoma*. In this context, the information available for human homologs can be used in comparative studies, at sequence and structure level, in order to understand the interactions of *Schistosoma* cystatins and host cysteine proteases. The present study aimed to identify cystatin homologs on predicted proteomes of three *Schistosoma* species and other Platyhelminthes in order to have a landscape view of the functional diversification in this phylum. In addition, evolutionary analyses were reconstructed for *Schistosoma* and human homologs based on the information at the sequence level, signatures, and phylogenetic relationships. Additionally, we evaluated cystatins' expression in different stages of the parasite life cycle in order to answer the following questions: How many cystatin homologs are present in *Schistosoma* species and in other Platyhelminthes? Do potential homologs have characteristic sequence features? What are the evolutionary relationships of the cystatin family members in *Schistosoma* species and their human homologs? Is the transcription of cystatin members during the *S. mansoni* life cycle stage-specific or is it conserved through the stages assessed?

In summary, we used predicted proteome data currently available for three *Schistosoma* species (Berriman et al., 2009; Zhou et al., 2009; Young et al., 2012), three Cestoda (Tsai et al., 2013), and the free living *Schmidtea mediterranea* (unpublished data) to identify potential cystatin homologs encoded by Platyhelminthes. Using combined computational approaches, we identified proteins belonging to the I25 family and reported members classified in two subfamilies: I25A and I25B. We also assessed microarray public datasets to investigate gene expression in different stages of the *Schistosoma mansoni* life cycle. This study provides insights into the evolution and potential functional diversification of Platyhelminthes cystatins improving our understanding of parasite biology and opening new frontiers in the identification of novel therapeutic targets against helminthiases.

## Materials and methods

### Organisms and sequence data

The dataset of selected species comprises three *Schistosoma* species: *S. haematobium* (NCBI taxid: 6185), *S. japonicum* (6182), and *S. mansoni* (6183); four other Platyhelminthes: *Echinococcus granulosus* (6210), *Hymenolepis microstoma* (85433), *Schmidtea mediterranea* (412041), and *Taenia solium* (6204); and *Homo sapiens* (9606). *Schistosoma* predicted proteomes were downloaded from SchistoDB 3.0 (beta.schistodb.net) (Zerlotini et al., 2013). Cestoda proteome data was obtained from the Sanger Institute FTP site (ftp.sanger.ac.uk/pub/pathogens). *S. mediterranea* proteome data was kindly provided by Dr. Eric Ross from Stowers Institute for Medical Research (USA). Predicted proteomes from each genome project were used in order to obtain evidence of protein gain or loss and a more accurate identification of cystatin homologs. *H. sapiens* I25 family members were retrieved from the Human Protein Reference Database (www.hprd.org) (Keshava Prasad et al., 2009). Functional information regarding the cystatin family is available on the MEROPS peptidase database (Rawlings et al., 2014) via the I25 inhibitor family identifier.

### Homologs identification

Potential cysteine protease inhibitors encoded by platyhelminth genomes were identified by using the hmmscan software included in the HMMER 3.0 package (Eddy, 2011). Each proteome was compared against Pfam-A HMM profiles, which were retrieved from the Pfam database (Finn et al., 2014). Such analyses were performed in order to identify the presence and architecture of proteins comprising the cystatin domain (Pfam: PF00031). The significance of the Pfam-A match is based on the resulting score. A match is considered significant when the score is greater than or equal to the gathering threshold for the Pfam domain. To date, the current threshold for the cystatin domain (Pfam: PF00031) is 20.9. Proteins containing significant or insignificant matches with the target domain were selected. Insignificant matches although less informative than significant ones, can be used for identifying functionally conserved regions when no significant matches are found. For this reason, insignificant matches were also initially selected in this work. In addition, information on accessory domains and protein signatures (Q-x-V-x-G motif, PW motif, LP motif, SND/SNS/TND motifs, and disulfide bonds) were considered to define potential I25 homologs. The presence of signal peptide in potential cysteine protease inhibitors was predicted by SignalP 4.1 using the neural network method with default D-cutoff values and using eukaryotes as “organism group” (Petersen et al., 2011). The illustrations of protein domain architectures were generated using DOG 2.0 (Ren et al., 2009).

### Phylogenetic analysis

Aiming at establishing evolutionary relationships among *Schistosoma* and experimentally validated human cystatins, I25A and I25B amino acid domain sequences from *S. haematobium*, *S. japonicum*, *S. mansoni*, and *H. sapiens* were selected for phylogenetic reconstruction. The evolutionary relationships between *Schistosoma* and human cystatins may provide cues about functions performed by parasites' orthologs. Human PF00031 domains classified into I25C subfamily or inhibitor units not assigned to a subfamily were not included in this analysis once they have no cysteine protease inhibitor activity. To optimize the dataset for phylogenetic analysis we removed redundancy and sequences too distantly related using the Decrease Redundancy tool, available as a resource at ExPaSy (www.expasy.org). The Decrease Redundancy parameters were set as 98 for “% max similarity” and 30 for “% min similarity.” The filtered set of amino acid sequences, corresponding to the conserved domain (PF00031) were aligned using MAFFT 7 with iterative refinement by the G-INS-i strategy (Katoh et al., 2009). The multiple sequence alignment comprising 22 sequences and 96 sites was manually refined using Jalview (Waterhouse et al., 2009) and further used in phylogenetic analysis. To reconstruct the phylogenetic tree we used MrBayes 3.2.1, which performs Bayesian inference using a variant of the Markov Chain Monte Carlo (Ronquist and Huelsenbeck, 2003). MCMC analyses were run as four chains, one cold and three heated chains, for 10,000,000 generations and sampled every 100 generations. Twenty-five percentage of the initial samples were discarded as “burn-in.” Mixed models were applied as a parameter to estimate the best-fit evolutionary model. Support values were estimated as Bayesian posterior probabilities. The evolutionary history of *Schistosoma* and human cystatins was also reconstructed based on the maximum likelihood method (ML), as implemented in PhyML (Guindon et al., 2010). For the phylogenetic reconstruction we tested 12 different evolutionary models (JTT, LG, DCMut, MtREV, MtMam, MtArt, Dayhoff, WAG, RtREV, CpREV, Blosum62, and VT) using the ProtTest 2.4 software (Abascal et al., 2005). The evolutionary model best fitting the data (best fit model) was determined by comparing the likelihood of the tested models according to the Akaike Information Criterion. Trees were visualized and edited using the FigTree software (tree.bio.ed.ac.uk/software/figtree).

### Transcriptional profiles

Data from 35,437 oligonucleotide microarray probes from *S. mansoni* transcriptomic analyses (Fitzpatrick et al., 2009) were interrogated in order to identify the transcription patterns of two cystatin family members: Smp_006390 (I25A) and Smp_034420.2 (I25B). Thirteen development stages were covered and the complete set of raw and normalized data were downloaded from ArrayExpress (https://www.ebi.ac.uk/arrayexpress/) under the experiment accession number E-MEXP-2094. For differential expression analysis, mean fluorescence normalized values were linear model fitted using three replicates per stage and a total of 19 evolutionary pairwise comparisons were made (see Fitzpatrick et al., 2009 for details). Additionally, recently published RNAseq transcription data (Protasio et al., 2012) was also interrogated for gene expression pattern and gene model evaluation. In this case, four developmental stages of *S. mansoni* were covered. Raw sequence datasets (three from cercariae stage, two from 3 h post-infection mechanically transformed schistosomula, two from 24 h post-infection schistosomulas, and one from adult worms), were downloaded from ArrayExpress under the accession number E-MTAB-451. The RNAseq reads were stored in a local server and aligned to the most recent version of the *S. mansoni* genome (v.5). Reads were mapped with Tophat-v.2.0.8 (Trapnell et al., 2012) and transcripts were assembled with Cufflinks-v.2.0.2 (Trapnell et al., 2012). Cuffdiff, a program from the Cufflinks suite, was used to estimate expression of transcripts across samples. CummeRbund, an R package, and Integrative Genomics Viewer -IGV (Thorvaldsdóttir et al., 2013) were used to visualize results.

## Results

In this study we have mined platyhelminth proteomes in order to identify proteins belonging to the I25 family and its respective subfamilies. To this end, we used intrinsic methods at sequence level followed by multiple sequence alignment and phylogenetic analysis. Such analyses generated an evolutionary view of potential cystatin proteins in three *Schistosoma* species. We also analyzed the amino acid sequence diversity focused on the identification of protein signatures. Finally, we verified the transcriptional profiles of cystatins. Overall, a framework for functional analysis of parasite cystatins is provided. In summary, our findings contribute to a better understanding of host-parasite interactions and pathogenesis, once analysis and cystatins appear as relevant molecules in these processes.

### Identification of cystatin family members

Cystatin family (I25) members were identified using an intrinsic method. Platyhelminth proteomes were scanned by hmmscan (Eddy, 2011) and potential homologs were retrieved based on the presence of significant or insignificant matches with the conserved cystatin domain (PF00031) (Table 1). In cases where insignificant PF00031 matches were recovered, we also searched for critical residues that mediate protease inhibition to define the query protein as a potential cysteine protease inhibitor (Table 2). Based on “start” and “end” alignment positions of potential homologs identified overlapping the PF00031 HMM profile, truncated regions were assigned. It is important to emphasize that the Pfam database (Finn et al., 2014) is built from the most recent UniProt (UniProt Consortium, 2014) release and that no single protein database covers all diversity existing in nature. More specifically, the total of platyhelminth cystatins available at UniProt is underrepresented when compared, for instance, to mammals. Thus, it is possible that the presence of divergent regions reflects their degree of divergence to other proteins available at the database. On the other hand, it is also important to consider that the difference between the PF00031 HMM profile and the query sequences can be related to the presence of pseudogenes or errors in the gene models.

**Table 1:** **Cystatin predictions across *Schistosoma* and other Platyhelminthes**.

**Taxon**	**TaxID**	**Accession**	**Length**	**Domain**	**Start**	**End**	***E*-value**	**Score**	**Significant**
*Schistosoma haematobium*	6185	Sha_109477	160	PF00031	83	142	2.2×10^−1^	11.6	No
		Sha_109478	145	PF00031	38	127	4.5×10^−2^	13.8	No
		Sha_300402	101	PF00031	35	91	1×10^−6^	28.7	Yes
*Schistosoma japonicum*	6182	Sjc_0005780	145	PF00031	37	126	1.2×10^−8^	35	Yes
		Sjc_0066340	101	PF00031	40	88	2.7×10^−7^	30.6	Yes
		Sjc_0094540	123	PF00031	40	85	1.8×10^−4^	21.5	Yes
*Schistosoma mansoni*	6183	Smp_006390.1	101	PF00031	35	91	1×10^−5^	25.5	Yes
		Smp_034420.1	117	PF00031	38	98	8.3×10^−7^	29	Yes
		Smp_034420.2	148	PF00031	38	129	7.9×10^−8^	32.3	Yes
		Smp_034420.3	145	PF00031	38	126	1.5×10^−2^	15.3	No
*Echinococcus granulosus*	6210	EgrG_000159200.1	98	PF00031	16	77	4.5×10^−5^	23.5	Yes
		EgrG_000159200.1	98	PF03672	29	51	2.2×10^−3^	17.5	No
		EgrG_000543900.1	111	PF00031	34	79	5.1×10^−3^	16.9	No
		EgrG_000849600.1	274	PF00031	45	98	2.3×10^−10^	40.4	Yes
		EgrG_000849600.1	274	PF00031	167	224	4.8×10^−2^	13.7	No
		EgrG_000849600.1	274	PF13549	37	104	1.1×10^−1^	11.7	No
*Hymenolepis microstoma*	85433	HmN_000582300.1	180	PF00031	41	83	2.4×10^−2^	14.7	No
		HmN_000582400.1	107	PF00031	26	78	1.5×10^−2^	15.4	No
		HmN_000582400.1	107	PF06050	18	51	4.4×10^−2^	12.5	No
		HmN_000842000.1	295	PF00031	63	115	3.2×10^−4^	20.7	No
*Taenia solium*	6204	TsM_000671000	274	PF00031	45	98	5.2×10^−10^	39.3	Yes
		TsM_000671000	274	PF00031	175	224	1.7×10^−2^	15.2	No
		TsM_000687900	98	PF00031	9	76	2.6×10^−4^	21	Yes
		TsM_000687900	98	PF03672	29	51	2.2×10^−2^	14.3	No
		TsM_000687900	98	PF13805	8	73	7.1×10^−2^	12.2	No
		TsM_001154200	115	PF00031	37	84	4×10^−3^	17.2	No
		TsM_001154200	115	PF14073	37	85	1.4×10^−1^	11.9	No
		TsM_001154200	115	PF15606	39	87	3×10^−2^	14.2	No
		TsM_001154300	137	PF00031	33	95	1.2×10^−4^	22.1	Yes
*Schmidtea mediterranea*	412041	mk4.000249.00.01	93	PF00031	4	51	8.6×10^−2^	12.9	No
		mk4.000249.04.01	93	PF00031	4	51	9.6×10^−2^	12.8	No
		mk4.004385.02.01	119	PF00031	33	104	1.3×10^−13^	50.8	Yes
		mk4.027397.00.01	176	PF00031	33	121	6.1×10^−14^	51.9	Yes

*TaxID: identifier at NCBI taxonomy database. Accession: accession number in the source genome project database. Length: number of amino acids. Domain: domain prediction based on HMM models from the Pfam database using the HMMscan tool. Start and End: alignment positions of the retrieved domain in the query sequence. Significant (Yes/No): statistically significant or insignificant score values according to the gathering threshold for each Pfam domain*.

**Table 2:** **Sequence features of I25 family members in selected taxa**.

**TaxID**	**Accession**	**SP**	**SND/SNS/TND**	**Q-x-V-x-G**	**S-S**	**LP**	**PW**
6185	Sha_109477	Yes	N/A	Yes	Yes	No	Yes
	Sha_109478	Yes	N/A	No	Yes	No	Yes
	Sha_300402	No	N/A	Yes	No	Yes	No
6182	Sjc_0005780	Yes	N/A	Yes	Yes	No	Yes
	Sjc_0066340	No	N/A	Yes	No	Yes	No
	Sjc_0094540	No	N/A	Yes	Yes	No	No
6183	Smp_006390.1	No	N/A	Yes	No	Yes	No
	Smp_034420.1	Yes	N/A	Yes	Yes	No	No
	Smp_034420.2	Yes	N/A	Yes	Yes	No	Yes
	Smp_034420.3	Yes	N/A	No	Yes	No	Yes
6210	EgrG_000159200.1	No	N/A	Yes	Yes	Yes	No
	EgrG_000543900.1	Yes	N/A	Yes	No	No	No
	EgrG_000849600.1_x	Yes	N/A	Yes	No	No	Yes
	EgrG_000849600.1_y	No	N/A	No	No	No	No
85433	HmN_000582300.1	No	N/A	Yes	No	No	No
	HmN_000582400.1	No	N/A	Yes	No	No	No
	HmN_000842000.1	No	N/A	Yes	No	No	No
6204	TsM_000671000_x	Yes	N/A	Yes	No	No	Yes
	TsM_000671000_y	No	N/A	No	No	No	No
	TsM_000687900	No	N/A	Yes	No	Yes	No
	TsM_001154200	Yes	N/A	No	No	No	No
	TsM_001154300	Yes	N/A	Yes	No	No	No
412041	mk4.000249.00.01	No	N/A	Yes	No	Yes	No
	mk4.000249.04.01	No	N/A	Yes	No	Yes	No
	mk4.004385.02.01	Yes	N/A	Yes	Yes	No	No
	mk4.027397.00.01	Yes	N/A	Yes	Yes	No	Yes
9606	O60676_CST8	Yes	No	No	Yes	No	Yes
	O76096_CYTF	Yes	Yes	Yes	Yes	No	Yes
	P01034_CYTC	Yes	Yes	Yes	Yes	No	Yes
	P01036_CYTS	Yes	No	No	Yes	No	Yes
	P01037_CYTSN	Yes	No	Yes	Yes	No	Yes
	P01040_CYTA	No	No	Yes	No	Yes	No
	P01042_KNG1	Yes	No	No	Yes	No	No
	P01042_KNG2	N/A	No	Yes	Yes	No	No
	P01042_KNG3	N/A	No	Yes	Yes	No	Yes
	P04080_CYTB	No	No	Yes	No	Yes	No
	P09228_CYTSA	Yes	No	Yes	Yes	No	Yes
	P28325_CYTD	Yes	No	Yes	Yes	No	Yes
	Q15828_CYTM	Yes	Yes	Yes	Yes	No	Yes
	Q5W186_CST9	Yes	No	No	Yes	No	No
	Q9H112_CST11	Yes	No	No	Yes	No	Yes
	Q9H114_CST1Like	Yes	Yes	No	Yes	No	Yes
	Q9H4G1_CST9Like	Yes	No	No	Yes	No	Yes

*TaxID: identifier at NCBI taxonomy database. 9606: Homo sapiens. Accession: accession number in the source genome project database or in UniProt (human proteins). Sequence features include a signal peptide (SP), disulfide bridge (S-S), and distinct motifs (SND/SNS/TND, Q-x-V-x-G, LP, and PW), in which amino acids are indicated by the one-letter code. Yes or No: presence or absence of conserved features. N/A: Not applicable*.

Considering alternative splicing products (Smp_034420.1, Smp_034420.2, and Smp_034420.3; Sha_109477 and Sha_109478), we identified in *Schistosoma* species ten proteins that contain the conserved domain (Table 1). Three proteins were retrieved in *S. haematobium*, three in *S. japonicum*, and four in *S. mansoni*. These single domain proteins vary in length and in domain size. In order to classify the identified homologs in subfamilies (I25A–C or unclassified), we searched for the presence of signal peptide and other evolutionarily conserved residues (Table 2), which are involved in the formation of a wedge-like structure that is complementary to the active site of target proteases. Features as Q-x-V-x-G, LP, and PW motifs which are considered essential for binding and inhibiting cysteine proteases activity were identified. To remove potentially redundant sequences as well as too distantly related proteins we filtered alternative splicing products and run the Decrease Redundancy program using the previously mentioned parameters. In total, four sequences were filtered out: Sha_109478, Sjc_0094540, Smp_034420.1, and Smp_034420.3.

Concerning other Platyhelminthes species, we identified 14 cystatin proteins encoded by three Cestoda (*E. granulosus*, *H. microstoma*, and *T. solium*) and a free living Turbellaria (*S. mediterranea*) (Table 1). Contrary to *Schistosoma* species, hmmscan searches retrieved additional domains in some of those homologs. Such domains were retrieved as insignificant matches and a few showed overlapping regions with the conserved domain (PF00031) (Table 1). The information of additional domains may suggest potential lineage-specific innovations that happened in cystatin family members over evolutionary time. On the other hand, it can reflect the caveat of data quality in organisms for which we have only draft genomes.

We also analyzed cystatin diversity at the sequence level in terms of critical motifs, amino acid conservation, or variants that could lead to differences in the inhibitory capability of the cystatins (Table 2 and **Figure 3**). The alignment of identified I25A and I25B cystatin sequences point out four conserved regions: a Glycine residue within the N-terminal region, a Q-x-V-x-G motif in one hairpin loop, and a PW or LP motifs in the second loop (**Figure 3**). Those regions can dock with the substrate-binding site of family C1 of cysteine proteases (Dickinson, 2002). One disulfide bridge exclusively present in I25B proteins was also identified. I25A subfamily members are predominantly intracellular single-domain proteins of about 11 kDa and ~100 amino acid residues, which do not contain disulfide bridges and the PW motif. I25A inhibitors have three evolutionarily highly conserved regions: a glycine residue within the N-terminal region, a central Q-x-V-x-G motif, and a C-terminal LP pair (Klotz et al., 2011).

**Figure 3** shows five *Schistosoma* and human homologs that have these conserved features, being therefore classified into the I25A subfamily. Following the same pattern of conserved features, when analyzing the proteome data of others Platyhelminthes (Table 2), we identified potential I25A subfamily member in *E. granulosus* (EgrG_000159200.1) and in *T. solium* (TsM_000687900). In *H. microstoma* three potential cystatin proteins without signal peptide were identified, something uncommon in other platyhelminth predictions. Therefore, this result should be further evaluated carefully, before being considered an evolutionary innovation. In *S. mediterranea* we identified two proteins belonging to the I25A subfamily. However the identified cystatins mk4.000249.04.01 and mk4.000249.00.01 are identical. For this reason we took into account the probability of redundancy and considered one of them (Table 2) as a cystatin homolog.

I25B inhibitors are secreted single-domain proteins around 14 kDa, ~120 residues long with at least one disulfide bridge and a signal peptide. I25B inhibitors have two of the three conserved regions previously mentioned: the N-terminal Gly residue and a central Q-x-V-x-G motif. Instead of a C-terminal LP pair, I25B inhibitors have a PW motif at the C-terminal segment. Besides, some I25B members also possess a distinct conserved SND, SNS or TND motifs between the first conserved glycine and the central Q-x-V-x-G motif (Table 2). The presence of these additional motifs allow cystatin proteins to inhibit either legumain or asparaginyl endopeptidases (Alvarez-Fernandez et al., 1999; Zavasnik-Bergant, 2008; Klotz et al., 2011; Schwarz et al., 2012). In parasites, I25B subfamily members were demonstrated to be involved in modulation of host immune responses (Khaznadji et al., 2005; Gregory and Maizels, 2008). **Figure 3** shows I25B *Schistosoma* and human sequences identified according the sequence features previously mentioned. In three Platyhelminthes species (*E. granulosus*, *H. microstoma*, *T. solium*) it was not possible to identify I25B homologs (Table 2). In *S. mediterranea* we identified two similar sequences. However, it seems like that mk4.004385.02.01 is a fragment of mk4.027397.00.01. In this case we have chosen the mk4.027397.00.01 protein as potentially true I25B homolog due to the presence of expected sequence features (Table 2).

I25C subfamily members act mostly on serine proteases classified into the family S8 (Cornwall et al., 2003) and metalloproteases from the family M12 (Valente et al., 2001). Fetuins and histidine rich proteins are also multi-domain secreted proteins, but lack cystatin activity and are called as unclassified (Rawlings et al., 2014). Kininogens and fetuins are much younger than I25A and I25B proteins and their occurrence are restricted to vertebrates (Kordis and Turk, 2009). According to our findings, the I25C and unclassified subfamily members are not encoded by the genomes of platyhelminth species here analyzed.

Human cystatin protein subunits previously characterized as protease inhibitors were classified as belonging to one of the three cystatin subfamilies 125A–C (Table 2). In total, 15 proteins were retrieved from the Human Protein Reference Database (Keshava Prasad et al., 2009) and evolutionarily conserved residues were identified (Table 2 and **Figure 3**). As already described in the literature, one additional motif was found (SND/SNS/TND), which is related to legumain inhibition. A multidomain protein P01042 was retrieved for each separate subunit denoted as KNG1, KNG2, and KNG3 (Table 2).

### Evolutionary relationships among *Schistosoma* and *H. sapiens* cystatins

The evolutionary relationships in cystatins were reconstructed from an alignment containing 22 sequences corresponding to the conserved domain (PF00031) and 96 sites (**Figure 3**) using maximum likelihood and Bayesian inference. Both methods retrieved the same tree topology. Statistical support was also calculated for each node by both phylogenetic inference methods (Bayesian inference/maximum likelihood). Protein sequences are represented on the phylogenetic tree by UniProt (UniProt Consortium, 2014) and SchistoDB (Zerlotini et al., 2013) identifiers. Based on the phylogeny we were able to identify two well supported monophyletic subfamilies (100/100): I25A and I25B (**Figure 4**). I25C and unclassified homologs were not identified in *Schistosoma* species. Two domains of the kininogen protein P01042 (KNG2 and KNG3) were placed in I25B clade due to its cysteine protease inhibitory activity. The KNG1 domain from the same protein belongs to the I25C subfamily and was not included in this analysis because it has lost its inhibitory activity due to mutations in structurally important regions (Kordis and Turk, 2009) and may act as a calcium transporter (Higashiyama et al., 1987).

According to the phylogeny (**Figure 4**), three *Schistosoma* proteins (Sha_300402, Sjp_0066340, and Smp_006390.1) and two human cystatin proteins (P04080_CYTB, P01040_CYTA) were grouped into the I25A subfamily clade. These proteins share the evolutionarily conserved residues: a N-terminal Gly, a central Q-x-V-x-G, and a C-terminal LP (Table 2 and **Figure 3**). Based on the information available on the literature and in protein databases, both human homologs (P04080_CYTB, P01040_CYTA) were experimentally and structurally characterized. On the other hand, only two I25A *Schistosoma* proteins (Smp_006390.1 and Sjp_0066340) were experimentally characterized at the protein level (Morales et al., 2004; He et al., 2011). Those proteins are involved in a intracellular modulator role. For instance, Smp_006390.1 was able to inhibit the formation of hemozoin by live schistosomula, suggesting a possible role in the gut of the schistosomula (Morales et al., 2004).

In I25B subfamily clade three *Schistosoma* homologs were grouped with 13 human proteins (14 cystatin domains). The conserved features (signal peptide, Gly residue, Q-x-V-x-G motif, disulfide bridge, and PW motif) were not detected in all protein domain sequences (Table 2 and **Figure 3**). Contrary to *Schistosoma* I25A subfamily members, I25B cystatins were not experimentally characterized. Human sequences placed on the I25B subfamily clade reveal a significant expansion of such subfamily in *H. sapiens*. The phylogenetic analysis shows that these homologs originated from successive post speciation gene duplication events. Most human I25B members present the typical protein signatures (Table 2 and **Figure 3**).

In summary, *Schistosoma* cystatin clades of I25A and I25B subfamily members were well supported by both branch support methods showing 100/100 (aLRT and posterior probability). *S. haematobium* and *S. mansoni* cystatins are closest related to each other when compare to the *S. japonicum* homolog. The phylogenetic analysis showed that human and *Schistosoma* cystatins are placed in different clades, reflecting its diversity at molecular level.

### Cystatin expression in *Schistosoma mansoni*

According to the microarray data analysis (Fitzpatrick et al., 2009), both I25A and I25B members, Smp_006390.1 and Smp_034420.2 respectively, were constitutively expressed in several stages of the *S. mansoni* life cycle (**Figure 5**). These findings corroborate the pairwise comparisons of key developmental stages performed by Fitzpatrick et al. (2009) that did not indicate any differential expression of both cystatin mRNAs. Comparisons were made with an adjusted *p* value (Adj *p* < 0.05), corrected using the Benjamini and Hochberg method for multiple testing, for which 3 replicates per life cycle were assessed. Both cystatins expression were also confirmed in the RNA sequencing work of Protasio et al. (2012). Transcripts were expressed constitutively in all four stages evaluated (data not shown). No differential expression assessment was considered in this case, mostly because those experiments did not include adult worm sample replicates. Therefore, without replicates, it is impossible to estimate sample variability.

As RNAseq data can be used to improve gene model annotations, current gene models for both I25A and I25B members were investigated. We analyzed the read coverage by mapping the reads against the reference genome. It was performed by Tophat, a tool that allows read alignments containing gaps in regions spanning introns. Therefore, predicted gene models for both cystatin family members were confirmed by visual inspection using IGV (Thorvaldsdóttir et al., 2013). All three exons of I25A and four exons of I25B, as well as both 5′ and 3′ UTR regions, located in the *S. mansoni* SuperContig 0138 and chromosome 2 respectively, had several reads correctly mapped within the exon limits (data not shown).

## Discussion

### Conserved sequence features in cystatins

The I25A cystatin subfamily is a predominantly intracellular protein and does not present disulfide bridges. The inhibitor domain Q-x-V-x-G is present in the first hairpin loop and contains the glycine residue in the first position of the cystatin domain. This amino acid is also conserved in other cystatins of the I25B subfamily (**Figure 3**). This glycine residue allows the N-terminal region to interact with the sub-sites S4, S3, and S2 of the cysteine proteases. In *Schistosoma* species, besides its role in degradation of hemoglobin, I25A can also act intracellularly as a general regulator of protease activity (Morales et al., 2004). The constant and ubiquitous expression in *S. mansoni*, as shown by transcriptomic analysis, supports this idea. In the I25A subfamily we identified the highly conserved LP motif in positions 73–74 (**Figure 3**). The LP motif is essential for high affinity binding to papain (Pol and Bjork, 1999). The conserved motif differences between I25A and I25B subfamilies could reflect differences in the inhibitory spectrum of these proteins during evolution of function (Dickinson, 2002).

**Figure 2:**
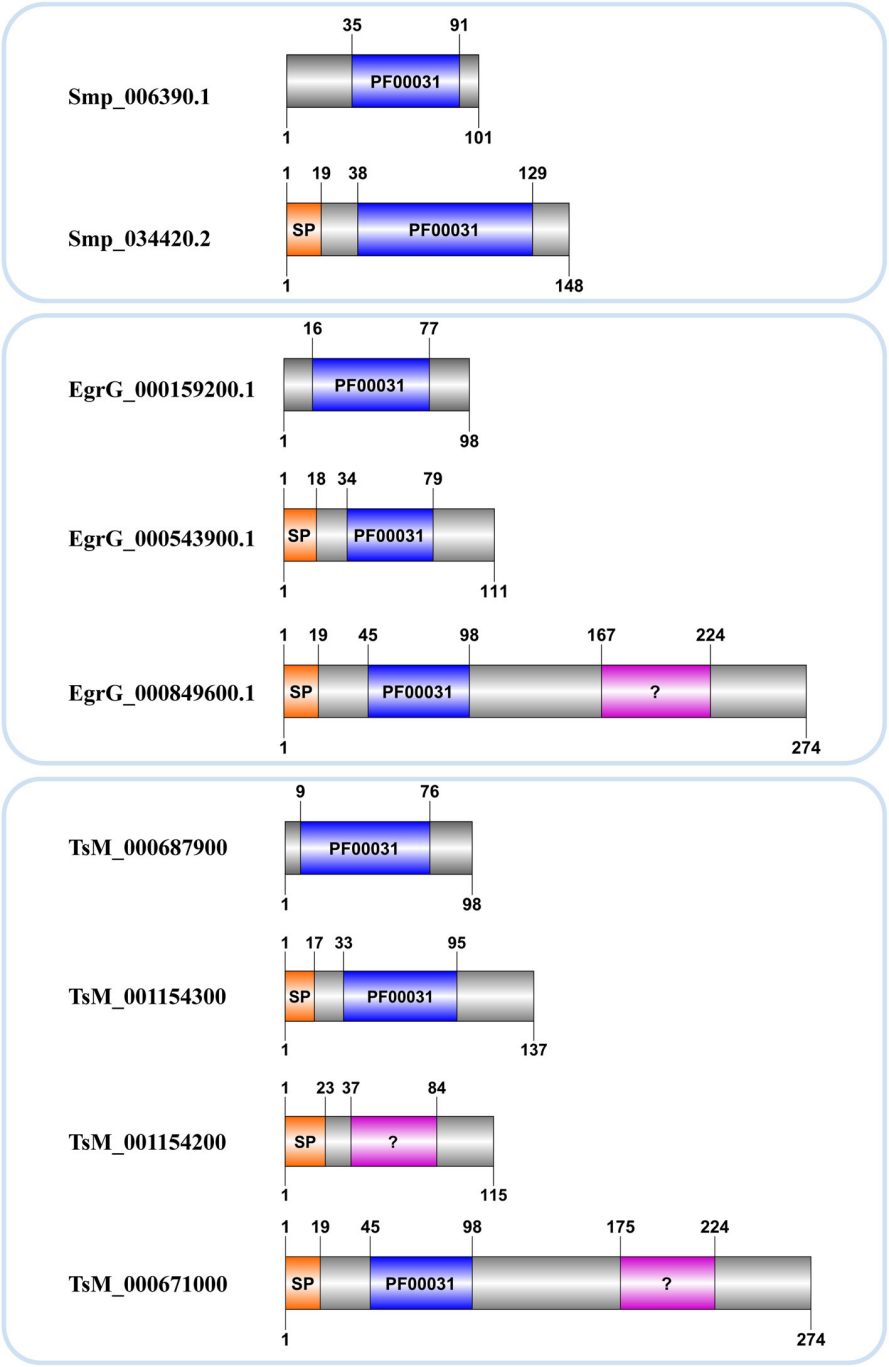
**Cystatin architecture in three Platyhelminthes**. Accession numbers correspond to those assigned in each genome project. Domain limits (above) and sequence length (below) are provided for each protein. A signal peptide (SP) and the conserved domain (PF00031) with significant (blue) or insignificant (?) matches are indicated.

Kordis and Turk (2009) postulated that the progenitor of this family was most probably intracellular, lacked a signal peptide and disulfide bridges. The hypothesis is that throughout cystatins evolution, gene duplications combined with deletions and insertions of genetic material resulted in single and multi-domain proteins with or without disulpfide bonds, glycosylated or not. Accordingly members of I25B subfamily likely evolved from I25A ancestors, which lack cysteine residues, acquiring disulfide bridges and signal peptide during evolutionary processes (Brown and Dziegielewska, 1997; Gregory and Maizels, 2008). In *Schistosoma* we identified a single I25B cystatin in each species, all of them containing critical motifs (Table 2). On the other hand, the *E. granulosus* protein EgrG_000849600.1 has signal peptide and two cystatin domains. One of them has an insignificant match with the PF00031 HMM profile (Table 2 and Figure 2). This multidomain protein did not show disulfide bridges, which is not typical for secreted proteins. The *T. solium* protein TsM_000671000 displayed architecture very similar to *E. granulosus* (Table 2 and Figure 2). Perhaps this indicates a protein architecture that is lineage-specific of Cestoda, although both of these species fall within the same cyclophyllidean family, and may not be representative of all tapeworms. The *H. microstoma* cystatins displayed an interesting protein architecture. They do not contain a signal peptide and also lack the LP motifs, unlike other I25A members (Table 2).

**Figure 3:**
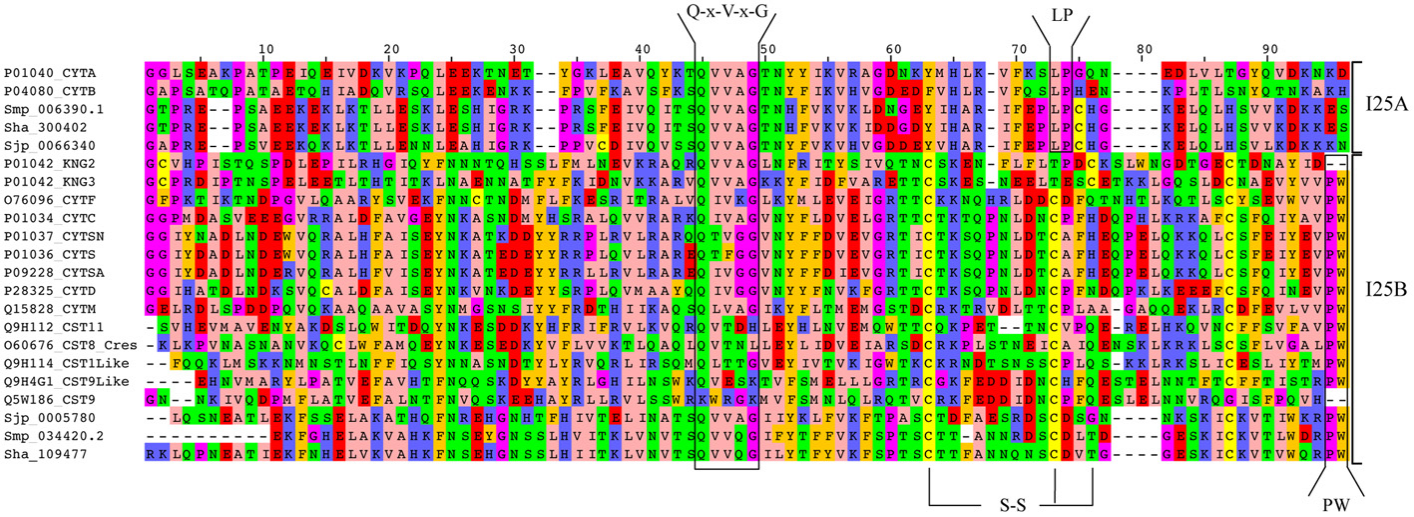
**Alignment of *Schistosoma* and human homologs**. Multiple sequence alignment of the conserved domain (PF00031) of I25A and I25B proteins of *S. haematobium* (Sha), *S. japonicum* (Sjp), *S. mansoni* (Smp), and *Homo sapiens* (UniProt accession numbers). Amino acid sequences were aligned using MAFFT with iterative refinement by the G-INS-i strategy. Conserved motifs (Q-x-V-x-G and PW) and disulfide bridges (S-S) are indicated.

The legumain inhibitory motifs (SND/SNS/TND) are distinct from the papain binding motif (Q-x-V-x-G) (Alvarez-Fernandez et al., 1999). These sites are located on the opposite side of the papain binding site (Gregory and Maizels, 2008) and were present in four human sequences P01034_CYTC (SND), Q15828_CYTM (SNS), O76096_CYTF (TND), Q9H114_CST1Like (SND) in position 28 of the cystatin domain (Table 2 and Figure 3). In *Schistosoma* and other Platyhelminthes, these motifs belonging to bifunctional cystatins were not present. However, in the nematode *Brugia malayi* the SND motif was identified in the secreted cystatin Bm-CPI-2 and was able to block the activity of mammalian legumain (Manoury et al., 2001). This inhibition profile is shared with other nematodes implying a dual function for nematode cystatins. In terms of adaptation to parasitism, Hartmann and Lucius (2003) compared I25B cystatins from filariae to those of *C. elegans*, and observed a distinct pattern of enzyme inhibitory activity and immunological properties.

Our results point to the diversity in terms of the presence and absence of sequence features used to classify cystatins (Table 2). An accurate cystatin classification based just upon these features is challenging. We used strict classification criteria, but we must take into account that many of the genomes here analyzed are still in their early versions and may contain inaccurate gene models, suggesting it is necessary to undertake manual curation for unambiguous annotation of cystatins and other proteins.

### Phylogenetic relationships in the cystatin family

**Figure 4:**
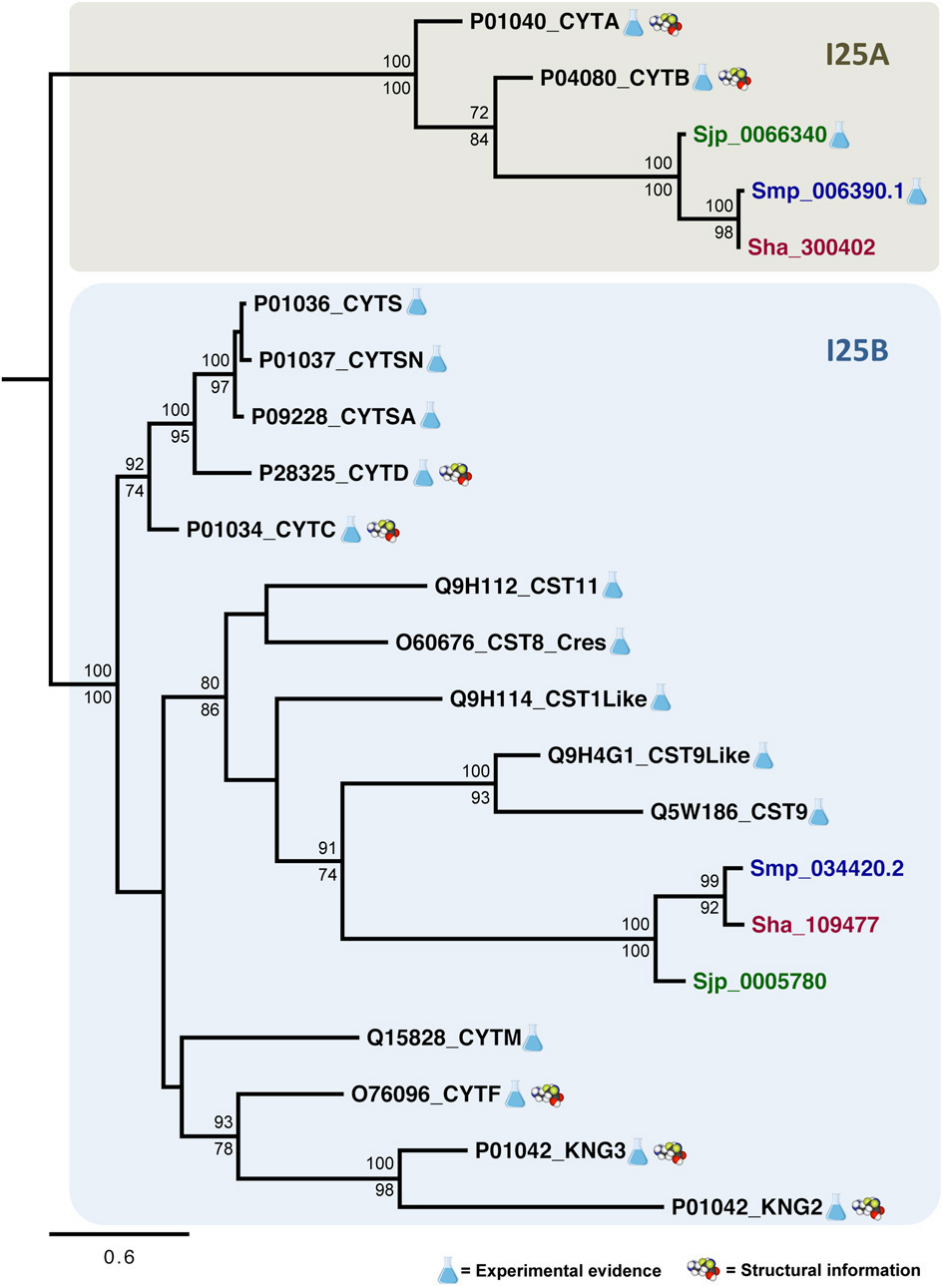
**Evolutionary relationships of I25A and I25B cysteine protease inhibitors**. A total of 22 amino acid sequences and 96 sites comprising the conserved domain (PF00031) of homologs encoded by *S. haematobium* (brown), *S. japonicum* (green), *S. mansoni* (blue), and *Homo sapiens* (black) were analyzed. The phylogeny was reconstructed by two methods using WAG was as the best fit model. In the Bayesian inference, support values for each node were estimated as posterior probability (above). In the maximum likelihood analysis, they were estimated using the Akaike Likelihood Ratio Test (aLRT) (below). Only support values higher than 70% are shown.

We identified two cystatin subfamilies, I25A and I25B, in the *Schistosoma*-Human phylogeny (Figure 4). In this work, a comparative analysis of *Schistosoma* cystatins and other 15 experimentally validated human cystatins belonging to diverse subgroups provided insights into the abundance, diversity, and evolution of *Schistosoma* cystatin family members. According to our results, the I25 cystatin family has a few members in *Schistosoma*, including I25A and I25B subfamilies' representatives in each species. Human cystatins have diversified significantly during the course of evolution, both at the sequence and functional levels, indicating that the cystatin domain is a protein-protein interaction module that can interact with novel targets (Alvarez-Fernandez et al., 1999; Dickinson, 2002; Abrahamson et al., 2003; Cornwall and Hsia, 2003; Kordis and Turk, 2009).

A subgroup in the I25B subfamily, named Cres (cystatin-related epididymal spermatogenic)/Testatin, was identified in our study with statistical support 80/86. This clade comprises I25B *Schistosoma* sequences. In human, glycoproteins of the Cres/Testatin subgroup are expressed in reproductive tissues and their function may be related to reproduction (Frygelius et al., 2010). The topology of this subgroup showed in Figure 4 is consistent with the phylogeny reported by Frygelius et al. (2010). Interestingly this subgroup lacks the consensus Q-x-V-x-G motif (Table 2 and Figure 3). Figure 4 shows that these proteins have a common origin and may represent a new subgroup within I25 family. Phylogenetic and comparative analyses show that genes involved in reproduction as Cres/Testatin and host pathogen interaction are under strong positive selection (Frygelius et al., 2010).

The phyletic distribution of the multidomain cystatins is limited and phylogenomic analyses suggest that multidomain cystatins are not monophyletic. Evidence suggests, they originated independently several times during evolution of eukaryotes (Kordis and Turk, 2009). Kininogen proteins (e.g., P01042) are multidomain and divergent cystatins containing three domains with different inhibitory properties. In our phylogenetic analysis we discarded the first domain as it lacks inhibitory activity (Rawlings et al., 2014). The two remaining kininogen domains (P01042_KNG2; P01042_KNG3) were placed in the I25B subfamily clade. The P01042_KNG2 and P01042_KNG3 domains contain the Q-x-V-x-G residues critical for inhibitory activity (Table 2). Both domains are grouped together with other human cystatins Q15828_CYTM and O76096_CYTF that inhibit both cysteine and asparaginyl protease due to the presence of the SNS and TND motifs, respectively (Table 2).

Our results suggest that *Schistosoma* species contain only two cystatin subfamilies, reflecting little functional diversification. Due to the presence of highly divergent sequences in I25B clade, the recognition of orthologous sequences is a difficult task. The intracellular cystatins belonging to I25A subfamily are more conserved than the divergent extracellular cystatins (I25B) (Figure 3), as reported for other proteins with similar features (Julenius and Pedersen, 2006).

Khaznadji et al. (2005) reported the first I25A multidomain protein in invertebrates, a multidomain I25A in the platyhelminth *Fasciola hepatica* containing six cystatin like domains, two of which are well conserved (Khaznadji et al., 2005). The intracellular and multidomain I25A inhibits parasite cathepsin L1 activity. The methods used by Khaznadji et al. (2005) to determine the domain architecture of this cystatin differs from those applied by MEROPS (Rawlings et al., 2014), which indicates the presence of a single domain in this protein. In addition, the presence of multidomain proteins are not the only novelty in the cystatin family, several I25A cystatins from unicellular eukaryotic organisms have gained the signal peptide, which is absent in the majority of metazoan and eukaryotic I25A cystatins. The presence of signal peptide as observed in some unicellular eukaryotic I25A cystatins (Kordis and Turk, 2009) and in *Fasciola gigantica* (Siricoon et al., 2012) may lead to the gain of novel defense functions.

In synthesis, the major obstacle to the identification and classification of cystatins using amino acid sequences is the fact that many of the proteins contain multiple homologous inhibitor domains in a single protein (Rawlings et al., 2014). Furthermore, phylogenetic analysis of cystatin family members is hampered by short protein length often added to the sequence divergence. In addition, different branches appear to have evolved at different rates (Dickinson, 2002).

### Cystatin expression

**Figure 5:**
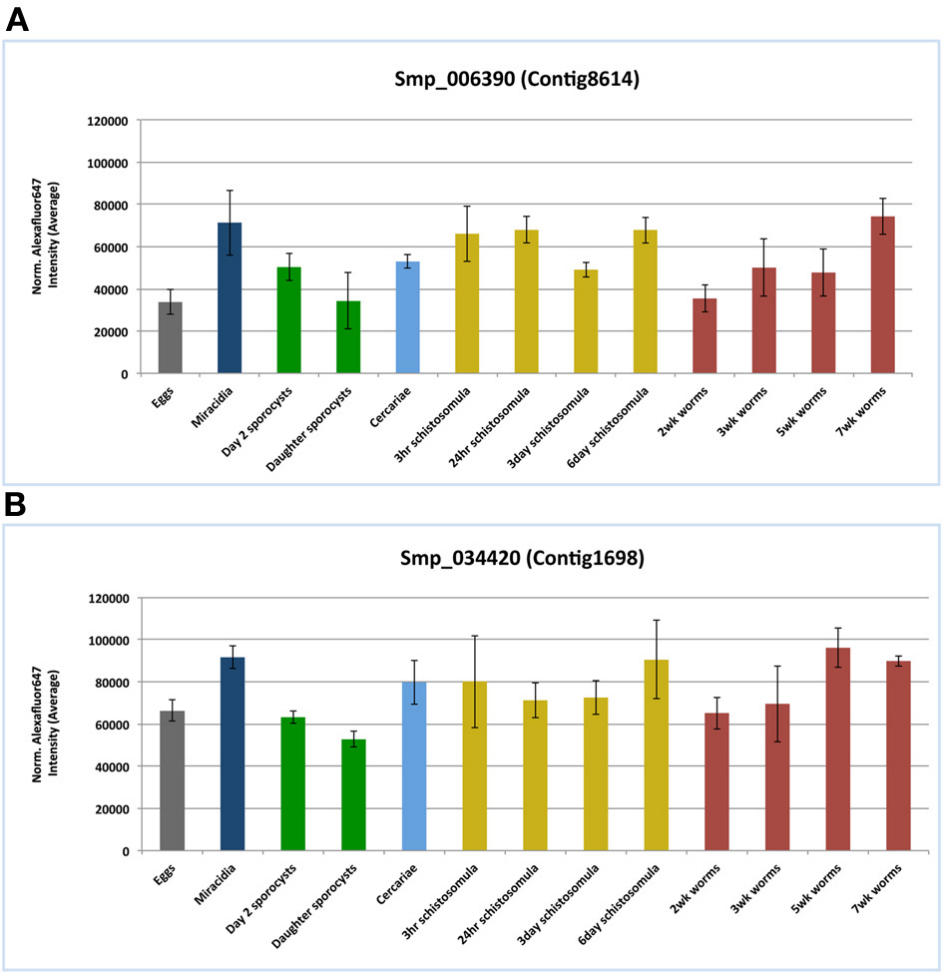
**Cystatins mRNA expression patterns in the *S. mansoni* life cycle**. Public microarray data available at ArrayExpress (E-MEXP-2094) was downloaded to a local server in order to identify cystatins expression patterns of the I25A subfamily member Smp_006390 **(A)** and the I25B subfamily member Smp_034420 **(B)** in *S. mansoni*. Bars correspond to the mean normalized values for each oligonucleotide probe named Smp_006390 and Smp_034420 in 13 different life stages.

In the present work, we interrogated publicly available gene expression datasets in order to investigate mRNA expression of both cystatin members I25A (Smp_006390.1) and I25B (Smp_034420.2) in *S. mansoni*. Microarray data by Fitzpatrick et al. (2009) assessed three ecological niches of *S. mansoni* life cycle (freshwater, molluscan, and definitive vertebrate host) and indicated similar expression levels of both cystatins among the parasite life cycle (Figure 5). The constitutive expression may be essential for schistosome life cycle progression. Published RNAseq data (Protasio et al., 2012) also point to the expression of cystatins in cercariae, schistosomula, and adults stages. Additional reports (Morales et al., 2004) suggest that I25A is expressed equally by adult males, females, and schistosomula stages. Therefore, both I25A and I25B are expressed throughout the *S. mansoni* life cycle.

In *S. japonicum*, He et al. (2011) observed not just the expression levels of the stefin Sjp_0066340, a I25A subfamily member, in egg, schistosomula, and adult stages by RT-PCR. He et al. (2011) also performed immunohistochemistry studies, which revealed that the *S. japonicum* stefin is mainly localized at the epithelial cells lining the gut as well as the tegument on the surface of adult worms. Additionally, the stefin of *Clonorchis sinensis* was also found mainly localized in the epithelial cells lining the intestine of the parasite (Kang et al., 2014). The stefin of *F. gigantica* was also localized in the intestinal epithelium and the tegumental type cell bodies together with the tegumental syncytium (Tarasuk et al., 2009). Altogether, the expression of parasite I25A proteins in the host-parasite interface point to a possible role in molecular interactions with host proteins, which are mostly inhibitors of host cysteine proteases such as cathepsins (Tarasuk et al., 2009; He et al., 2011; Kang et al., 2014).

Recently, an unusual secreted form of I25A member was characterized in *F. gigantica* (Siricoon et al., 2012). Although this awkward cystatin does present a signal peptide, typical of I25B proteins, sequence analysis does correlate it to the I25A subfamily. Nevertheless, Siricoon et al. (2012) provided evidence of a secreted form of a cystatin protein in Platyhelminthes that was again observed in the intestinal epithelium in all developmental stages. Moreover, the secreted 125A protein was also found expressed in the prostate gland in the adult stage of *F. gigantica*, which suggests a regulative role of cysteine protease activity in reproductive system. Similarly, the expression of human cystatin I25B subgroup proteins, also called Cres/Testatin, was localized at the reproductive tissues and their function may be related to reproduction (Frygelius et al., 2010). Interestingly, human Cres/Testatin subgroup was placed in the same clade with the *Schistosoma* cystatins I25B (Figure 4).

Based on the evidence of expression in related organisms and given the constitutive expression of cystatins I25A and I25B in *S. mansoni*, it is possible that cystatin functions can be involved in key processes in *Schistosoma*. Such proteins may be required to keep its proteolytic activity balanced as well as to protect the parasite against degradation by host or endogenous proteins. Nevertheless, cystatin tissue-specific expression, such as those identified at the reproductive system in human and *F. gigantica*, could evidence a more specialized role against specific cysteine proteases.

## Conclusions

In summary, our evolutionary analysis using genomic, transcriptomic, and proteomic data for three *Schistosoma* species and other Platyhelminthes has provided the first insights into the evolution, classification, and functional diversification of platyhelminth cystatins. These findings improve our understanding concerning the diversity, at the molecular level, of cystatins encoded by such species. Only two subfamilies (I25A and I25B) were clearly identified in *Schistosoma* and Platyhelminthes reflecting the low diversification of this family when compared to human. Regarding Cestoda, it is necessary to implement an exhaustive study in order to better understand the domain composition revealed in our work.

We expect that this study will encourage experimental and structural characterization of cystatins in *Schistosoma* and other closely related parasites. Altogether, studies involving parasite cystatins will help to elucidate the functions performed by those proteins as well their correlation with parasite biology and host-parasite interaction. The importance of new insights revealed by functional genomics as RNAi experiments and comparative expression patterns across different life cycle stages in *Schistosoma* and other Platyhelminthes will provide a functional landscape of the cystatin role in the parasite life cycle.

## Author contributions

Conceived and designed the experiments: Yesid Cuesta-Astroz, Larissa L. S. Scholte, Fabiano Sviatopolk-Mirsky Pais, Guilherme Oliveira, and Laila A. Nahum. Carried out homologs and protein signatures identification: Yesid Cuesta-Astroz and Larissa L. S. Scholte. Performed expression analysis: Fabiano Sviatopolk-Mirsky Pais and Yesid Cuesta-Astroz. Performed the phylogenetic studies: Yesid Cuesta-Astroz and Larissa L. S. Scholte. Wrote the manuscript: Yesid Cuesta-Astroz, Larissa L. S. Scholte, Fabiano Sviatopolk-Mirsky Pais, Guilherme Oliveira and Laila A. Nahum. Reviewed and revised the manuscript: Laila A. Nahum and Guilherme Oliveira. Coordinated this study: Laila A. Nahum and Guilherme Oliveira. All authors have read and approved the final manuscript.

### Conflict of interest statement

The Guest Associate Editor Arnon Dias Jurberg declares that, despite being affiliated to the same institution as authors Cuesta-Astroz, Scholte, Pais, Oliveira and Nahum, the review process was handled objectively and no conflict of interest exists. The authors declare that the research was conducted in the absence of any commercial or financial relationships that could be construed as a potential conflict of interest.
